# Knowledge, Attitude, and Practice of Emergency Department Healthcare Professionals Towards Traumatic Dental Injuries Across Public Hospitals in Riyadh, Saudi Arabia

**DOI:** 10.3390/dj14030154

**Published:** 2026-03-09

**Authors:** Haifa AlAmro, Asma Alshahrani, Kiran Iyer, Latifah Almashabi, Hala Alanazi, Arwa Alshahrani, Nouf Alrawaf

**Affiliations:** 1 Department of Preventive Dental Sciences, College of Dentistry, King Saud bin Abdulaziz University for Health Sciences, Riyadh 14611, Saudi Arabia; shahranias@ksau-hs.edu.sa (A.A.); iyerk@ksau-hs.edu.sa (K.I.); 2 King Abdullah International Medical Research Center, Riyadh 11481, Saudi Arabia; 3 Ministry of National Guard-Health Affairs, Riyadh 11426, Saudi Arabia; 4 College of Dentistry, King Saud bin Abdulaziz University for Health Sciences, Riyadh 14611, Saudi Arabia

**Keywords:** traumatic dental injuries, emergency department healthcare professionals, public hospitals, dental trauma, physician, dentist, nurse

## Abstract

**Background/Objectives**: Emergency department healthcare professionals are often the first to provide treatment, yet studies indicate limited knowledge in Traumatic Dental Injuries (TDIs) management among these providers. While research on TDI management in emergency departments exists globally, recent data from Saudi Arabia is lacking. This study aims to assess the knowledge, attitude, and practice of emergency department physicians, nurses, and Emergency Medical Services (EMS) regarding TDIs in three major hospitals in Riyadh, Saudi Arabia. **Results**: Physicians (46.4%) were significantly (*p* = 0.02) more likely to respond that they have sufficient knowledge about TDIs compared to nurses and EMS personnel. Of the 221 respondents, 136 (62%) were in the age group of 30–40 years, while females (OR 4.23, *p* = 0.00, CI 2.29–23.13) and nurses (OR 4.88, *p* = 0.00, CI 2.39–9.96) were more likely to say ‘No’ for any form of training they received in dental trauma during their education. Young (20–30 years old) professionals (OR 3.70, *p* = 0.04, CI 1.53–8.92) were less likely to feel confident in managing cases of dental trauma compared to their senior colleagues. **Conclusions**: In this study, nurses’ and EMS personnel’s knowledge of dental trauma management was poorer than that of physicians. Most respondents reported that dental trauma management was not part of their curriculum or training, which, in turn, was reflected in their low confidence in managing such cases.

## 1. Introduction

Traumatic dental injuries (TDIs) are frequently encountered in emergency departments and constitute a prevalent oral health problem among pediatric and adolescent populations. If TDIs were included in the world’s most frequent diseases, it could rank fifth [1]. About 40% of children experience a TDI to their primary teeth, and up to one-third of children sustain trauma to their permanent teeth [2,3,4,5]. There have been reports that the frequency of visits to emergency rooms for dental care has increased [6,7,8]. The severity level of TDIs can range from minimal enamel cracks to complex fractures that expose the dental pulp, and from subluxation to complete avulsions, with permanent teeth avulsion accounting for 0.5–16% of all dental injuries [9,10,11,12]. TDIs can be caused by a variety of factors, such as contact sports, playground accidents, falls, motor vehicle accidents, or even eating hard and stiff foods. It has been well documented that the prognosis of a traumatized tooth is largely dependent on both the timing and adequacy of emergency management, especially avulsions [13]. If treatment is delayed, the patient may experience a poorer prognosis for the involved teeth [14].

Emergency dental cases, particularly severe traumatic injuries, are more frequently encountered in after-hours emergency care settings within both public and private healthcare institutions [15,16]. Emergency physicians, nurses, and Emergency Medical Services (EMS) are often the first responders to provide primary treatment. Studies have shown that emergency physicians have limited knowledge and skills in the management of TDIs [17,18,19,20,21]. The necessity of expanding the undergraduate medical curriculum to incorporate the emergency care of TDIs was further confirmed by a study looking at the fundamental knowledge and attitudes of medical students in Saudi Arabia [22]. In addition, a study carried out in Canada indicated that Emergency Department (ED) physicians feel relatively unprepared by their residency training to treat dental complaints, and professional dental support is an issue in the majority of EDs [23]. A review of medical doctors’ knowledge of dental trauma management had also found an overall deficiency in the knowledge and confidence in managing TDIs [24]. Among different ED healthcare professionals, a study carried out in Saudi Arabia revealed that EMS had the least knowledge of severe oral injuries, whereas surgeons had the best understanding of them [25]. Physicians’ confidence in dental trauma management was also surveyed among 285 participants, with only 19% being confident in treating such cases [26].

Emergency department healthcare professionals must possess comprehensive knowledge of the assessment and management of TDIs. Although several studies have been published worldwide, there is a paucity of recent research examining the knowledge level among different ED personnel. Therefore, this study aims to assess the knowledge, attitude, and practice of TDIs among emergency department physicians, nurses, and EMS personnel in three major public hospitals in Riyadh, Saudi Arabia.

## 2. Materials and Methods

This study followed the STROBE guidelines for reporting methodology of cross-sectional studies.

### 2.1. Study Design

A descriptive, cross-sectional study was conducted to assess the knowledge, attitude, and practice of healthcare professionals actively involved in emergency patient care, including emergency physicians, nurses, and EMS personnel.

### 2.2. Study Setting

Data collection for this study was conducted from January 2025 to June 2025. Before the commencement of the study, Institutional Review Board (IRB) approval was obtained (NRR24/068/8) from King Abdullah International Medical Research Center (KAIMRC). Prior to the distribution of the self-administered questionnaire, informed consent was obtained from all participants after a detailed explanation of the study. Participants were assured that their contribution was voluntary, their responses were anonymous, and that no personal identifiers were collected.

### 2.3. Participants

This study adopted the non-probability, convenience sampling technique for participants being enrolled from three major public hospitals in Riyadh, Saudi Arabia, each recognized by their high-capacity emergency departments managing dental trauma cases. The study included participants involved in the emergency healthcare services at these public hospitals. Exclusion criteria included individuals who did not provide informed consent, as well as those who were not involved as first responders in trauma cases and consequently did not participate in the management of TDIs.

Sample size estimation was performed using G Power software (Heinrich-Heine-Universität, Düsseldorf, Germany, version 3.1.9.4). At alpha 0.05 with an effect size of 0.3 and power of 0.95, the total sample size was determined to be 220.

### 2.4. Questionnaire

The questionnaire was adopted from previously published scientific papers on the topic [17,18,19,20,21], with some modifications. Prior to data collection, the questionnaire was assessed for content validity by quantitative analysis of expert judgments using Aiken’s V assessment [27]. Eight experts from the specialty of pediatric dentistry and endodontics provided input on the questionnaire. Experts rated each question on a predefined ordinal scale, and Aiken’s V values were calculated. All questions scored between 0.9 and 1, and thus were found to be valid, except for one question, which scored 0.7 and was removed from the questionnaire. Data were collected using a Google Forms-based questionnaire that was distributed with the assistance of station supervisors at the participating public hospitals. It was administered in person using iPads and QR codes to facilitate access and completion.

The questionnaire was divided into two sections: demographic characteristics and knowledge, attitude, and practice (KAP). The demographic section comprised five questions representing the independent variables. The KAP domains constituted the dependent variables and included five questions each assessing knowledge and attitude, and three questions assessing practice. Knowledge-related questions evaluated awareness of TDIs, guidelines, and clinical principles; attitude-related questions assessed confidence, satisfaction, and willingness to engage in further learning; and practice-related questions examined exposure to dental trauma cases and management-related behaviors. Greater emphasis was placed on teeth avulsion as it represents one of the most time-critical TDIs, in which prompt and appropriate emergency management is strongly associated with improved prognosis.

### 2.5. Statistical Analysis

The data from Google Forms was exported to Microsoft Excel (Microsoft Corp., New York, NY, USA) for data cleaning and coding purposes and later analyzed using the Statistical Package for the Social Sciences (SPSS) statistical software (VERSION 20, 2011; IBM Corp., Armonk, NY, USA). Descriptive statistics were used to describe all the qualitative variables based on frequency and percentage. Pearson’s chi-square analysis was used to find significant differences in knowledge, attitude, and practice with exploratory variables. Significant variables based on chi-square analysis were explored further with multinomial regression analysis.

## 3. Results

A total of 230 responses were collected; however, nine were excluded because they were social workers and were not involved in the management of TDIs. The final analysis included 221 valid responses. The majority (62%) of the participants were in the 30–40-year age group. The response rates for males (48%) and females (52%) were found to be similar. Among the respondents, 146 (66%) had 0–5 years of work experience. A high frequency of physicians (50%) responded to the questionnaire among all emergency medical personnel, followed by nurses (38%) and EMS personnel (Table 1).

An assessment of the highest-frequency responses to each of the questions indicated that the respondents in this study felt their knowledge levels were insufficient with respect to TDIs. Interestingly, a considerable number of these personnel in the emergency departments responded that they rarely (69, 31.2%) come across dental trauma in the department. More than half of the respondents (124, 56.1%) indicated that they had not received any form of training in the management of TDIs (Table 2).

There was a 50% likelihood of respondents indicating that they had observed tooth avulsion. Thirty-three percent of the respondents would refer to the dentist if they came across an avulsed tooth, with almost 39% choosing to refer ‘immediately’, indicating the necessity for immediate dental management. Among the options for preserving the tooth before the dentist could attend or could be reached, the majority of these personnel reported that storing the avulsed tooth in sterile saline would be the best method. The type of tooth (permanent or primary) did influence the decision-making of replanting the tooth, according to the respondents: among the 172 (77.8%) who answered ‘yes’, the decision making varied, as most of them (140, 63.3%) considered permanent teeth to be important compared to the primary teeth 15 (6.8%) (Table 2).

The independent variables gender, age, hospital, and profession significantly influenced the response, as observed in the chi-square analysis. Gender-based variation in response was elicited for the following questions: “In your experience, how often do patients present with dental trauma at your emergency department?” and “Did you receive training on the management of dental trauma during your education?” The latter question exhibited that females (41.7%) were more likely (*p* = 0.00) to respond with ‘rarely ‘compared to the males. More interestingly, a significant (*p* = 0.00) contrasting response was observed for the former question regarding training received, with the majority of males (51.9%) responding ‘yes’ compared to their female counterparts. For the question “If you came across a child with a ‘knocked out’ (avulsed) tooth, what would you do?” the females were more likely (36.5%) to consider the option “Wash the child’s mouth with water and put the tooth in a wet cloth”; in contrast, 31.1% of male participants preferred referring immediately to a dentist (Table 3).

Personnel employed in the ERs of the three public hospitals varied significantly in their responses to two of the questions, namely “In your experience, how often do patients present with dental trauma at your emergency department?” and “Did you receive training on the management of dental trauma during your education?”. For the latter question, a significant (*p* = 0.01) number of personnel (45.1%) from hospital 3 reported observing TDIs ‘rarely’ in their emergency department compared to responses to the same questions from public hospital 1 (25.9%) and public hospital 2 (23.1%) (Table 4).

With regard to two knowledge-based questions and two practice-based questions, there was significant variation in responses among the EMS personnel, nurses, and physicians. Fifty-one Physicians (46.4%) were significantly (*p* = 0.02) more likely to respond that they had sufficient knowledge of TDIs compared to the EMS personnel and nurses; likewise, 52 physicians (47.3%) were significantly (*p* = 0.00) more likely to mention that they had received training on managing TDIs during their education (Table 4).

Multinomial Regression analysis was carried out for all independent variables found to be significant against the responses in the chi-square analysis. There were significantly higher odds of females (OR 1.57, *p* = 0.01, CI 1.21–5.45) and nurses (OR 2.44, *p* = 0.03, CI 1.08–5.53) reporting that in their experience, patients ‘rarely’ presented with dental trauma at their emergency departments; similarly, females (OR 4.23, *p* = 0.00, CI 2.29–23.13) and nurses (OR 4.88, *p* = 0.00, CI 2.39–9.96) were more likely to answer ‘No’ to the question on receiving any form of training in dental trauma during their education. In regard to encountering an avulsed tooth, females (OR 0.23, *p* = 0.01, CI 0.94–0.57) were significantly less likely than males to put the tooth back in the socket and refer to the dentist; their inclination was more towards washing the mouth and putting the tooth in wet gauze, though this was not statistically significant. Compared to other professions, EMS personnel were significantly (OR 7.50, *p* = 0.04, CI 1.03–54.11) more likely to ‘throw the tooth and just try to stop the bleeding’ when they encountered an avulsed tooth (Table 5).

The age of the professionals had a significant impact on their experience with an event such as avulsion due to trauma; the likelihood of younger (20–30 years old) professionals (OR 3.86, *p* = 0.00, CI 2.03–7.32) answering ‘no’ was significantly higher than that of the other two age groups of 30–40 years and more than 40 years of age. Likewise, younger (20–30 years old) professionals (OR 3.70, *p* = 0.04, CI 1.53–8.92) were less likely to feel confident in managing cases of dental trauma compared to the other two age groups; similarly, they felt their knowledge levels were not satisfactory (OR 0.55, *p* = 0.77, CI 0.28–1.06), which contrasted with findings from the other two older age groups (Table 5).

## 4. Discussion

Traumatic dental injuries constitute genuine dental emergencies that demand immediate clinical assessment and, when indicated, urgent intervention, particularly in cases of permanent tooth avulsion. Although initial first-aid measures for TDIs are typically straightforward and cost-effective, the accuracy and timeliness of emergency management play a decisive role in determining long-term prognosis. Conversely, inadequate knowledge of such emergencies may impede accurate clinical judgment and delay appropriate treatment. Such injuries are commonly encountered in emergency department settings, where the initial evaluation is frequently undertaken by ED personnel.

This study evaluated the knowledge, attitude, and practice of emergency department healthcare professionals, including physicians, nurses, and EMS staff, regarding the management of traumatic dental injuries in three major public hospitals in Riyadh, Saudi Arabia. The findings reveal significant knowledge gaps and variations in confidence levels among different professional groups, consistent with regional and international findings [20,21,22,23,24,25,26,28,29].

In the present study, more than half of the respondents reflected insufficient knowledge regarding the management of traumatic dental injuries. This finding is consistent with other studies that reported a lack of knowledge among emergency medical staff in regard to the management of TDIs [17,28,30,31,32], with percentages as high as 90% [21]. Moreover, multiple studies have highlighted significant deficiencies in emergency physicians’ and nurses’ knowledge of TDI management, with particular concern regarding the appropriate treatment of avulsed teeth [31,32,33]. The International Association of Dental Traumatology recommends that avulsed teeth be managed through proper storage and preservation until replantation is possible, immediate replantation when circumstances permit, and subsequent stabilization with splinting followed by endodontic therapy under the care of a dentist [34].

A clear deficiency in knowledge regarding the appropriate storage media was also observed, as 30% of the respondents chose sterile saline as the most suitable storage medium. Sterile saline lacks metabolically essential ions, does not provide glucose to the cells, and damages the tooth cells within a short time [35,36]. Other studies have also reported that participants were not familiar with the most suitable storage medium [20,22,30,31,37], which in turn affected the prognosis of the avulsed tooth. Another crucial aspect of replantation of the avulsed tooth is that this approach is only recommended for permanent teeth; primary teeth should not be replanted to avoid further damage to the succedaneous teeth. In the present study, 22% of the respondents stated that the type of avulsed tooth (primary vs. permanent) would not affect their decision to replant the avulsed tooth. This has also been observed in a study by Cully et al., in which respondents were confused about whether the injured teeth were primary or permanent [26]. Moreover, immediate replantation of an avulsed permanent tooth was selected as the correct response by 26.4% of physicians, followed by 11.1% of EMS personnel, and only 2.4% of nurses.

Confidence in managing TDIs varied among respondents, with 41.6% being very confident, although 42% reported insufficient knowledge, and 56% had no previous training in such cases. Cully et al. reported similar findings of respondents’ confidence [26], in contrast to other studies with low confidence [17,38].

The study examined key factors that could potentially influence knowledge, attitude, and practice in managing dental trauma among healthcare professionals. The results revealed several significant associations that highlight gaps in awareness and training across genders, professions, and age groups. Male participants reported higher exposure to training in TDI management and were more likely to encounter dental trauma cases in their respective emergency departments compared with female participants. Evidence from existing literature suggests that studies restrict their reporting on gender-based responses broadly to knowledge and attitude towards dental trauma management [39,40]. Based on the present findings, gender appears to be an important variable that merits consideration in future research examining practice-related differences among healthcare professionals.

As respondents’ age increased, the likelihood of witnessing accidents involving an avulsed tooth also increased. Participants aged 20–30 years were significantly less likely to have encountered patients requiring avulsion management in their emergency rooms. A study by Baginska J et al. reported a direct correlation between experience among nurses and their likelihood of encountering tooth avulsion []. The study also stated that they were more likely to respond better to the questions; this aspect was also corroborated by findings from the study by Doğan, Ö et al., who assessed the knowledge and skills of paramedics regarding dental trauma [40]. Another finding of Doğan, Ö et al. was that the majority of their study population was dissatisfied with their knowledge of dental trauma, which corroborates the findings of the present study, especially given that the dissatisfaction was evident in the younger age group (20–30 years).

Nurses were found to be significantly less likely than physicians to have received training in dental trauma management, followed by EMS personnel. This disparity underscores the urgent need for interdisciplinary trauma education, as it was found that nurses had a weaker understanding of dental trauma procedures compared to physicians and emergency healthcare professionals. This echoes the findings of Coşkun et al., who also highlighted the need for interdisciplinary trauma education through seminars, case discussions, and continuing educational programs [30].

The influence of specialty and level of experience on TDI knowledge remains a subject of debate in the literature [17,18,19,21,32,37,41]. Nonetheless, studies have highlighted that practitioners from disciplines with greater exposure to TDIs tend to demonstrate superior performance [17,19,20]. In the present study, 46% of physicians, 30% of nurses, and 40% of EMS personnel rated their knowledge of TDI management as sufficient. These variations may be partly explained by differences in prior training, as 47% of physicians, 15% of nurses, and 44% of EMS personnel reported having received formal education or training in TDI management. Additionally, variability observed among the three hospitals may be attributable to differences in orofacial trauma patient volume, institutional protocols for dental trauma management, and the diverse educational backgrounds of emergency healthcare professionals, many of whom received their training in different countries.

When respondents were asked about the importance of establishing an educational program on TDI management, 90% affirmed its necessity, and 80% indicated that they would be willing to participate in such training if made available. This finding is consistent with findings from a study by Wolfer et al., where recommendations were also given to include TDI management training for emergency room personnel [20]. Other recommendations in the literature included establishing guidelines and aids, such as “Dental Trauma Guide”, and the adoption of a decision-making pathway to provide timely management, improve emergency personnel comfort, and enhance outcomes for pediatric patients presenting with a dental trauma [20,26]. Another recommendation is to enhance access to simulation-based training. In Saudi Arabia, Khattab et al. noted that some academic hospitals have begun integrating simulation into their educational programs [42]. Supporting this, Zafar et al. found that the use of dental trauma simulation training offers an additional means of learning and has the potential to be used as an adjunct tool in the learning and management of dental traumatology. The majority of their participants felt more prepared to treat patients presenting with traumatic dental injury in the future, and 93% agreed/strongly agreed that it added value to their training compared to receiving only traditional didactic instruction [43].

While the present findings are broadly consistent with previous studies, this study contributes new evidence by providing updated, region-specific data related to TDI management among emergency department healthcare professionals in Riyadh, Saudi Arabia. Importantly, it offers a comparison between physicians, nurses, and EMS personnel, which has been limited in prior research. The current findings emphasize the need for targeted educational strategies tailored to specific professional groups.

The present study has several limitations. First, the potential for unintentional selection bias cannot be overlooked due to the voluntary nature of the survey. Voluntary participation may have contributed to the higher levels of knowledge observed, as participants who chose to respond were likely those with a greater interest in or familiarity with dental trauma, thereby potentially inflating the reported knowledge levels. Although the survey was distributed across three major hospitals and included participants from diverse clinical backgrounds, the sample may not be fully representative of the broader healthcare population, as the number of respondents may not adequately capture the full diversity of experiences, training levels, and exposure to dental trauma across all clinical settings. Additionally, variation in educational level, both between and within professional groups, was not assessed in the present study and warrants consideration in future investigations. Furthermore, as the study was conducted in only three public hospitals in Riyadh, Saudi Arabia, the findings may not be generalizable to the national healthcare context and should be interpreted as being limited to the participating institutions. Future studies incorporating both public and private healthcare facilities at the national level would strengthen the external validity of the findings.

## 5. Conclusions

• Emergency department healthcare professionals demonstrated insufficient training and suboptimal preparedness in the management of dental trauma.
• Nurses and EMS personnel showed lower levels of knowledge compared with physicians.
• Considerable variability was observed in participants’ exposure to dental trauma training during their professional education.
• Dental trauma appears to receive limited emphasis in the curricula of non-dental healthcare professions.
• Targeted educational interventions, including focus groups, may improve competence and confidence in the management of dental trauma among emergency care providers.

## Figures and Tables

**Table 1:** Descriptive analysis of each of the demographic profiles of respondents.

Demographic Variable	Sub-Division/ Categories	Total (N)	Frequency	Percentage (%)
Age Group of Participants	20–30	221	22	10
	30–40		136	62
	>40		63	28
Gender	Male	221	106	48
	Female		115	52
Years of Experience	0–5 years	221	146	66
	6–10 years		43	19
	11–20 years		24	11
	>20 years		08	04
Hospital Currently Working at	Public Hospital 1	221	85	39
	Public Hospital 2		65	29
	Public Hospital 3		71	32
Profession of Participants	(EMS) personnel	221	27	12
	Nurse		84	38
	Physician		110	50

**Table 2:** Descriptive analysis of each of the KAP domain questions pertinent to dental trauma management.

Domain	Question	Categories	Total (N)	Frequency	Percentage (%)
**Knowledge**	How do you rate your knowledge in dental trauma management	Sufficient	221	87	39.4
		Insufficient		93	42.0
		I have no knowledge		41	18.6
	Did you receive training on the management of dental trauma during your education?	Yes	221	77	34.8
		No		124	56.1
		Do not remember		20	9.0
	How would you keep the tooth until the patient reaches the dentist?	Any septic solution	221	6	2.7
		Does not matter		9	4.1
		I don’t know		19	8.6
		In a dry cloth, gauze		16	7.2
		In patient’s saliva		12	5.4
		Milk		61	27.6
		Special nutrient medium		12	5.4
		Sterile saline		67	30.3
		Tap water		10	4.5
		Wet handkerchief		9	4.1
	Does the type of tooth (adult or baby) affect your decision to replant (put it back in its socket)?	Yes	221	172	77.8
		No		49	22.2
	If yes, which tooth would you consider to replant?	Both	221	14	6.3
		Answered no in the previous question		49	22.2
		None		3	1.4
		Permanent (adult) tooth		140	63.3
		Primary (baby) tooth		15	6.8
**Attitude**	How urgent do you feel that a dentist’s opinion is needed in such cases?	Before the next day	221	12	5.4
		Immediate		86	38.9
		Not needed		7	3.2
		Within 30 min		60	27.1
		Within a few hours		56	25.3
	What is your level of confidence in managing/treating dental emergencies?	Very confident	221	18	8.1
		Somewhat confident		92	41.6
		Somewhat unconfident		62	28.1
		Very unconfident		49	22.2
	Do you think it is important to have an educational program in dental trauma management?	Yes	221	198	89.6
		No		23	10.4
	If a dental trauma management training was given, would you attend?	Yes	221	176	79.6
		No		45	20.4
	How satisfied are you with your knowledge on the management of dental trauma?	Extremely Satisfied	221	16	7.2
		Satisfied		107	48.4
		Unsatisfied		79	35.7
		Extremely Unsatisfied		19	8.6
**Practice**	In your experience, how often do patients present with dental trauma at your emergency department?	Weekly	221	51	23.1
		Monthly		57	25.8
		Every 2–3 months		37	16.7
		Rarely		69	31.2
		Never		7	3.2
	Have you come across an accident where a tooth was ‘knocked out’ (avulsed)?	Yes	221	111	50.2
		No		110	49.8
	If you came across a child with a ‘knocked out’ (avulsed) tooth, what would you do?	I don’t know	221	22	10.0
		Put the teeth back into the socket and rush to the dentist		34	15.4
		Refer immediately to a dentist		73	33.0
		Throw the tooth and just try to stop the bleeding		23	10.4
		Wash the child’s mouth with water and put the tooth in a wet cloth		69	31.2

**Table 3:** Chi-square analysis of independent variables (gender and age of respondents) with that of responses.

Question	Gender	n (%)					Total		df	Chi-Square Test (*p*-Value)
		Weekly	Monthly	Every 2–3 Months	Rarely	Never				
In your experience, how often do patients present with dental trauma at your emergency department?	Female	24 (20.9)	26 (22.6)	15 (13.0)	48 (41.7)	2 (1.7)	115 (100)		4	0.00 *
	Male	27 (25.5)	31 (29.2)	22 (20.8)	21 (19.8)	5 (4.7)	116 (100)			
	Total	51 (23.1)	57 (25.8)	37 (16.7)	69 (31.2)	7 (3.2)	221 (100)			
Did you receive training on the management of dental trauma during your education?	Gender	n (%)					Total		2	0.00 *
		Do not remember		No		Yes				
	Female	15 (13.0)		78 (67.8)		22 (19.1)	115 (100)			
	Male	5 (4.7)		46 (43.4)		55 (51.9)	106 (100)			
	Total	20 (9.0)		124 (56.1)		77 (34.8)	221 (100)			
If you came across a child with a ‘knocked out’ (avulsed) tooth, what would you do?	Gender	n (%)						Total	4	0.01 *
		I don’t know		Put the teeth back into the socket and rush to the dentist	Refer immediately to a dentist	Throw the tooth and just try to stop the bleeding	Wash the child’s mouth with water and put the tooth in a wet cloth			
	Female	13 (11.3)		9 (7.8)	40 (34.8)	11 (9.6)	42 (36.5)	115 (100)		
	Male	9 (8.5)		25 (23.6)	33 (31.1)	12 (11.3)	27 (25.5)	106 (100)		
	Total	22 (10.0)		34 (15.4)	73 (33.0)	23 (10.4)	69 (31.2)	221 (100)		
Have you come across an accident where a tooth was ‘knocked out’ (avulsed)?	Age	n (%)					Total		2	0.00 *
		Yes		No						
	20–30	51 (37.5)		85 (62.5)			136 (100)			
	30–40	44 (69.8)		19 (30.2)			63 (100)			
	>40	16 (72.7)		6 (27.3)			22 (100)			
	Total	111 (50.2)		110 (49.8)			221 (100)			
What is your level of confidence in managing/treating dental emergencies?	Age	n (%)					Total		6	0.02 *
		Very confident		Somewhat confident	Somewhat unconfident	Very unconfident				
	20–30	11 (8.1)		45 (33.1)	44 (32.4)	36 (26.5)	136 (100)			
	30–40	4 (6.3)		37 (58.7)	14 (22.2)	8 (12.7)	63 (100)			
	>40	3 (13.6)		10 (45.5)	4 (18.2)	5 (22.7)	22 (100)			
	Total	92 (41.6)		62 (28.1)	18 (8.1)	49 (22.2)	221 (100)			
How satisfied are you with your knowledge on the management of dental trauma?	Age	n (%)					Total		6	0.03 *
		Extremely satisfied		Satisfied	Extremely unsatisfied	Unsatisfied				
	20–30	10 (7.4)		55 (40.4)	17 (12.5)	54 (39.7)	136 (100)			
	30–40	5 (7.9)		37 (58.7)	1 (1.6)	20 (31.7)	63 (100)			
	>40	1 (4.5)		15 (68.2)	1 (4.5)	5 (22.7)	22 (100)			
	Total	16 (7.2)		107 (48.4)	19 (8.6)	79 (35.7)	221 (100)			

* Significance: *p*-value (<0.05) and df (degree of freedom).

**Table 4:** Chi-square analysis of independent variables (associated hospital and profession) with that of responses.

Question	Hospital	n (%)										Total	df	Chi-Square Test (*p*-Value)
		Every 2–3 Months	Monthly		Never		Rarely			Weekly				
In your experience, how often do patients present with dental trauma at your emergency department?	Hospital 1	19 (22.4)	27 (31.8)		1 (1.2)		22 (25.9)			16 (18.8)		85 (100)	8	0.01 *
	Hospital 2	10 (15.4)	16 (24.6)		5 (7.7)		15 (23.1)			19 (29.2)		65 (100)		
	Hospital 3	8 (11.3)	14 (19.7)		1 (1.4)		32 (45.1)			16 (22.5)		71 (100)		
	Total	37 (16.7)	57 (25.8)		7 (3.2)		69 (31.2)			51 (23.1)		221 (100)		
Did you receive training on the management of dental trauma during your education?	Hospital	n (%)										Total	2	0.02 *
		Yes				No								
	Hospital 1	81 (95.3)				4 (4.7)						85 (100)		
	Hospital 2	59 (90.8)				6 (9.2)						65 (100)		
	Hospital 3	58 (81.7)				13 (18.3)						71 (100)		
	Total	198 (89.6)				23 (10.4)						221 (100)		
How do you rate your knowledge in dental trauma management?	Profession	n (%)										Total	4	0.02 *
		Sufficient		Insufficient					I have no knowledge					
	EMS Personnel	11 (40.7)		13 (48.1)					3 (11.1)			27 (100)		
	Nurse	25 (29.8)		35 (41.7)					24 (28.6)			84 (100)		
	Physician	51 (46.4)		45 (40.9)					14 (12.7)			110 (100)		
	Total	87 (39.4)		93 (42.1)					41 (18.6)			221 (100)		
In your experience, how often do patients present with dental trauma at your emergency department?	Profession	n (%)										Total	8	0.04 *
		Weekly	Monthly		Every 2–3 months			Rarely			Never			
	EMS Personnel	9 (33.3)	4 (14.8)		3 (11.1)			10 (37)			1 (3.7)	27 (100)		
	Nurse	15 (17.9)	15 (17.9)		16 (19)			34 (40.5)			4 (4.8)	84 (100)		
	Physician	27 (24.5)	38 (34.5)		18 (16.4)			25 (22.7)			2 (1.8)	110 (100)		
	Total	51 (23.1)	57 (25.8)		37 (16.7)			69 (31.2)			7 (3.2)	221 (100)		
Did you receive training on the management of dental trauma during your education?	Profession	n (%)										Total	4	0.00 *
		Yes		No					Do not remember					
	EMS Personnel	12 (44.4)		13 (48.1)					2 (7.4)			27 (100)		
	Nurse	13 (15.5)		61 (72.6)					10 (11.9)			84 (100)		
	Physician	52 (47.3)		50 (45.5)					8 (7.3)			110 (100)		
	Total	77 (34.8)		124 (56.1)					20 (9)			221 (100)		
If you came across a child with a ‘knocked out’ (avulsed) tooth, what would you do?	Profession	n (%)										Total	8	0.00 *
		I don’t know	Put the teeth back into the socket and rush to the dentist		Refer immediately to a dentist			Throw the tooth and just try to stop the bleeding			Wash the child’s mouth with water and put the tooth in a wet cloth			
	EMS Personnel	2 (7.4)	3 (11.1)		4 (14.8)			6 (22.2)			12 (44.4)	27 (100)		
	Nurse	10 (11.9)	2 (2.4)		27 (32.1)			13 (15.5)			32 (38.1)	84 (100)		
	Physician	10 (9.1)	29 (26.4)		42 (38.2)			4 (3.6)			25 (22.7)	110 (100)		
	Total	22 (10)	34 (15.4)		73 (33)			23 (10.4)			69 (31.2)	221 (100)		

* Significance-*p* value (<0.05), and df (degree of freedom).

**Table 5:** Multinomial regression analysis of significant independent variables compared to dependent variables.

Variable	Response	Independent Variable	B	df	Sig.	Exp(B)	95% Confidence Interval for Exp(B)	
							Lower Bound	Upper Bound
In your experience, how often do patients present with dental trauma at your emergency department?	Rarely	Female	0.94	1	0.01 *	1.57	1.21	5.45
		Male	0#	-	-	-	-	-
	Rarely	EMS Personnel	0.18	1	0.73	1.20	0.41	3.43
		Nurse	0.89	1	0.03 *	2.44	1.08	5.53
		Physician	0#	-	-	-	-	-
Did you receive training on the management of dental trauma during your education?	No	Female	1.44	1	0.00 *	4.23	2.29	23.13
		Male	0#	-	-	-	-	-
	No	EMS Personnel	0.11	1	0.78	1.12	0.46	2.70
		Nurse	1.58	1	0.00 *	4.88	2.39	9.96
		Physician	0#	-	-	-	-	-
If you came across a child with a ‘knocked out’ (avulsed) tooth, what would you do?	Put the teeth back into the socket and rush to the dentist	Female	−1.46	1	0.01 *	0.23	0.94	0.57
		Male	0#	-	-	-	-	-
	Throw the tooth and just try to stop the bleeding	EMS Personnel	2.01	1	0.04 *	7.50	1.03	54.11
		Nurse	1.17	1	0.10	3.25	0.78	13.48
		Physician	0#	-	-	-	-	-
Have you come across an accident Where Tooth was ‘knocked out’ or avulsed?	No	20–30 years	1.35	1	0.00 *	3.86	2.03	7.32
		>40 years	−0.14	1	0.79	0.86	0.29	2.56
		30–40 years	0#	-	-	-	-	-
What is your level of confidence in managing/treating dental emergencies?	Very Unconfident	20–30 years	1.30	1	0.04 *	3.70	1.53	8.92
		>40 years	0.83	1	0.21	2.31	0.61	8.63
		30–40 years	0#	-	-	-	-	-
How satisfied are you with your knowledge on the management of dental trauma?	Satisfied	20–30 years	−0.59	1	0.77	0.55	0.28	1.06
		>40 years	0.48	1	0.41	1.62	0.51	5.11
		30–40 years	0#	-	-		-	-

# Category considered as the reference group. B—Odds. Exp(B) exponential Odds. * *p* < 0.05—Significant.

## Data Availability

The original contributions presented in this study are included in the article. Further inquiries can be directed to the corresponding author.
